# Identification of the fibroin of *Stigmaeopsis nanjingensis* by a nanocarrier-based transdermal dsRNA delivery system

**DOI:** 10.1007/s10493-022-00718-7

**Published:** 2022-05-11

**Authors:** Xia Li, Rundong Liu, Gang Li, Daochao Jin, Jianjun Guo, Ronald Ochoa, Tianci Yi

**Affiliations:** 1 Institute of Entomology, Guizhou University, Guiyang, 550025 China; 2 Guizhou Provincial Key Laboratory for Agricultural Pest Management of the Mountainous Region, Guiyang, 550025 China; 3 Scientific Observing and Experimental Station of Crop Pest in Guiyang, Ministry of Agricultural and Rural Affairs, Guiyang, 550025 China; 4 Systematic Entomology Laboratory (SEL), Agricultural Research Service (ARS), Beltsville Agricultural Research Centre (BARC), United States Department of Agriculture (USDA), Maryland, 20705 USA

**Keywords:** *Stigmaeopsis nanjingensis*, Silk, Fibroin, RNAi, Nanocarrier

## Abstract

*Stigmaeopsis nanjingensis* (Ma and Yuan) (Acari: Tetranychidae) is an important pest of bamboo—feeding behavior and silk production by the female adult mites is seriously harmful to bamboo leaves. Due to its small size, silking and cocooning, its management is difficult. This study discusses a fast and easy method for management of the pest by disturbing the spinning behavior. *Stigmaeopsis nanjingensis* is host specific and feeds only on bamboo leaves. Leaf margins of bamboo are highly hydrophobic, which makes dsRNA difficult to immerse. Hence, it is a challenge to apply the commonly used feeding method to inhibit gene expression in mites. In this study, we deliver dsRNA to interfere with the expression of fibroin by body wall permeation with a nanocarrier-based delivery system. The dsRNA/nanocarrier formulation droplets could enter the body cavity within 2 min after falling on the mite. The fibroin silencing efficiency was 75.4%, and the results of electron microscopy showed that dsRNA/nanocarrier damage the morphological structure of the silk thread. This study demonstrated the effectiveness of a nanocarrier-based percutaneous dsRNA delivery system in *S. nanjingensis* and its effect on the fibroin gene that influences the spinning behavior of *S. nanjingensis.* These findings may provide a new delivery system for RNAi-based control of spider mites that utilize protective webbing in the field.

**Supplementary Information:**

The online version contains supplementary material available at 10.1007/s10493-022-00718-7.

## Introduction

Silk is produced by a wide variety of arthropods (Hoffman et al. 1996) including Arachnida and Myriapoda (Craig et al. 1997) and more than 20 insect orders, such as Hymenoptera, Lepidoptera and Neuroptera. Silk plays an important role in the life history, growth, foraging, nesting, breeding, and metamorphosis of arthropods (Sutherland et al. 2010). Insect silk is composed of fibroin, sericin, external wax and various impurities. Fibroin is secreted and synthesized by posterior silk glands of insects and is the main component of arthropod silk. Fibroin is a natural biological macromolecule with no physiological activity and is insoluble in water. It is mainly composed of low-complexity dipeptide repeating units composed of Gly-Ala or Gly-Ser (Zhou et al. 2001). According to the conformation of the fibroin molecular chain, the structures of silk are mainly divided into three types: α-helices, β-folds, and random coils.

At present, research on fibroin focuses mostly on silkworm (Song 2002). In 1972, Suzulki isolated the fibroin mRNA of the posterior silk gland of *Bombyx mori*, and identified part of its sequence (Zhou et al. 2000). Subsequently, the full sequences of 13 genes containing silk fibroin were obtained from the posterior silk gland, and some of these genes were cloned (Ohshima and Suzuki 1977). During physical chromatogram analysis, it was found that the 5′ end of the silk fibroin gene was rich in AT (Manning et al. 1978). Then, its function and regulation were extensively studied. Research showed that the transcription factor *trx* (Li 2017), *E2F* and *Ras1* gene play a regulatory role in the synthesis of silk proteins, the tyrosine kinase gene Abl has a certain effect on silk protein secretion (Liu 2018), and miR-2805 significantly downregulate the expression of *B. mori* fibroin light-chain gene (Chen 2019). Studies have also been conducted on *Sylepta derogata*, a main pest for cotton and another cash crop, the larvae of which cause damage by spinning silk and leaf rolling. The fibroin of *S. derogata* was cloned in 2016, RNAi was used to interfere with the expression of fibroin and affect the silking behavior (Chen 2018).

In Acari, *Tetranychus* spp. are typical species that spin silk for dispersal and movement between plants (Clotuche et al. 2011). However, at present, analysis of spider mite silk is limited. Seventeen genes of *Tetranychus urticae* Koch were annotated by very weak sequence similarity with fibroin genes of other arthropods, encoding fibroin with an abnormally high serine content (27–39%) (Grbić et al. 2011). In 2020, *Tetranychus lintearius* was shown to produce a large amount of nanosized silk, and fluorescence labelling showed that this silk was a new cytocompatible material and a potential source of natural nanoparticles with various potential applications (Lozano-Perez et al. 2020). Through comparative genomics and multigroup analysis, two kinds of fibroin were identified and showed a high Young's modulus (Arakawa et al. 2021), even exceeding that of spider silk, showing that spider mite silk has considerable application prospects.

**Fig. 1:**
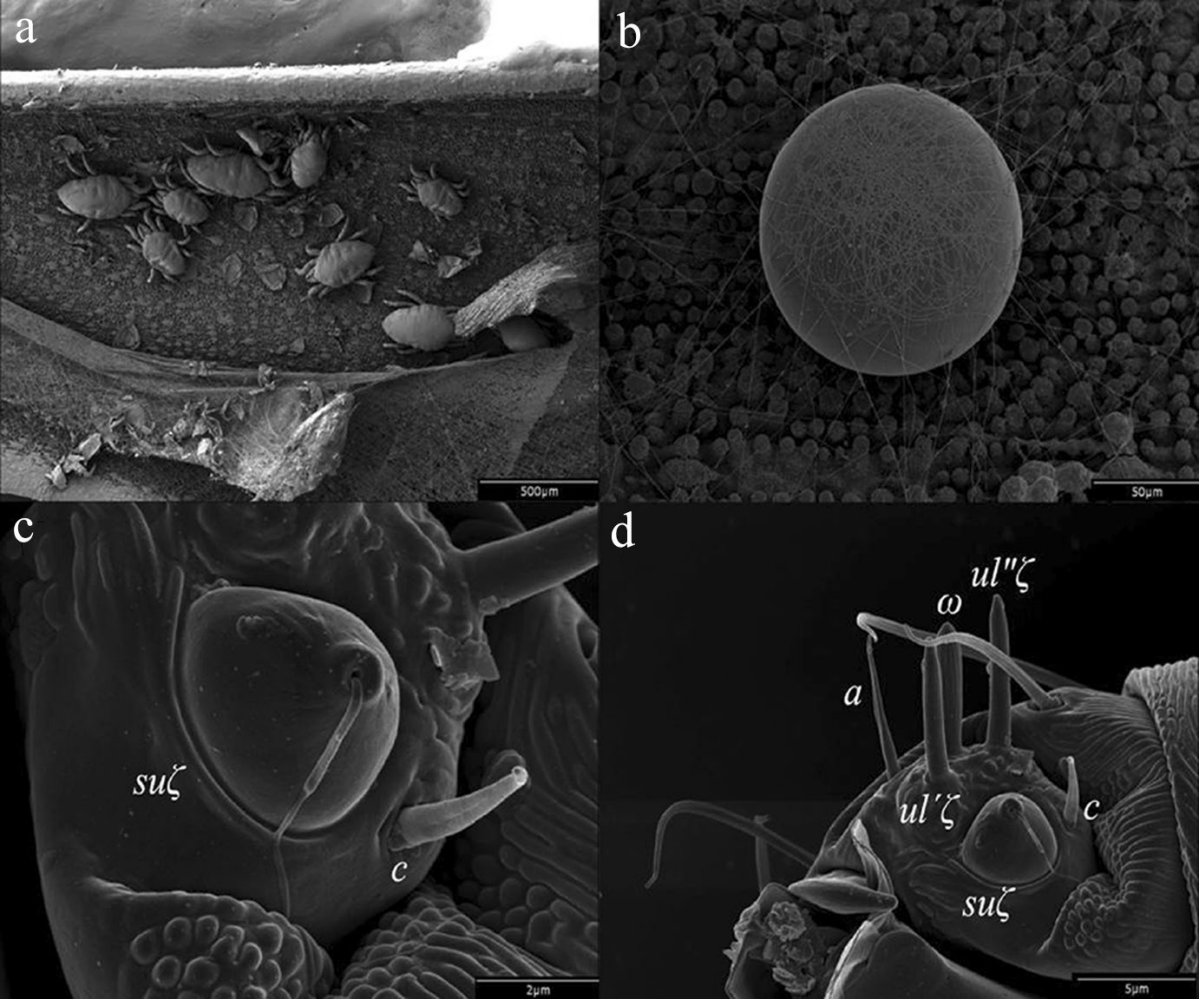
Electron micrographs of female *Stigmaeopsis nanjingensis* and her spinnerets. **a** Colony of *S. nanjingensis*, after manual removal of the woven net top*.* (**b**) Web mat woven for fixing eggs. **c** and **d** Spinnerets of *S. nanjingensis* at **c** 13,000× and **d** 5000×. *suζ*: terminal eupathidium; *ul′ζ* and *ul″ζ*: two lateral eupathidia; *ω*: solenidion; a, c: two tactile setae

*Stigmaeopsis nanjingensis* belongs to the family Tetranychidae and can produce a large amount of silk; the function of its silk is different from that of the other spider mites. *Stigmaeopsis nanjingensis* is a main pest of bamboo, and uses dense silk threads to make nests (Pellizzari and Duso 2009) (Fig. 1a). Both male and female mites have silk spinneret (Fig. 1c, d), but only female adult mites can weave nests. In the nests, female mites weave sticky silk and stick waste residue to the top, so as to clean the nest; Male mites cooperate to maintain their nests. In addition, *S. nanjingensis* weaves a web mat around the eggs to fix their position to prevent them from being disturbed during mite activities (Kanazawa et al. 2011) (Fig. 1b). These are strong indicators that silk is essential for *S. nanjingensis* growth and development. *Stigmaeopsis nanjingensis* caused severe damage to bamboo leaves during high-temperatures season from July to August that led to plant dieback due to falling of leaves. Under these circumstances, it is difficult to provide relief via biological and chemical control. The design of the nest can reduce the risk of predation (Mori et al. 1999), and being located on the underside of the leaves makes the control of *S. nanjingensis* more complicated. Therefore, we provide a control measure from the perspective of interfering with the silking behavior of *S. nanjingensis*. However, a systematic study of the fibroin genes expressed in *S. nanjingensis* has not been reported yet. Here, we report the transcriptome of *S. nanjingensis* and verify one of the fibroin genes.

RNAi is a post-transcriptional gene-silencing mechanism (Hutvangner and Zanmore 2002). RNAi with double-stranded RNA (dsRNA) has been used for analysis of gene functions in arthropods (Wilkins et al. 2005). Although the RNAi system was first established for insects, its application in mite research is common now. Several studies mainly focused on basic gene function analysis and screening of lethal target genes (Niu et al. 2018). Several methods have been developed for the delivery of dsRNA, including external contact, injection and feeding (Suzuki et al. 2017a, b; Guang et al. 2019). However, no research has been carried out on dsRNA transmission in *S. nanjingensis* to date. Due to the small size of the mites and the risks of physical damage or infection, injection is relatively complex. The soaking method consumes a large amount of dsRNA. Feeding is the most commonly used method for spider mites, such as *Tetranychus urticae* (= *cinnabarinus*) (Shen et al. 2017; Yoon et al. 2018) and *Amphitetranychus viennensis* (Zacher) (Zhao 2020). *Stigmaeopsis nanjingensis* is known to occur only on Gramineae plants (Sakagami et al. 2009), and bamboo is its main host. Leaf structure of bamboo makes dsRNA transmission difficult to enter the leaves for the feeding of mites. As the leaf develops, it forms two different regions of wettability which persists as the leaf bends down into its natural position (Wigzell et al. 2016). Leaf margins are highly hydrophobic whereas the middle of the leaf is hydrophilic in nature. Liquid landing on the leaf margins forms beads which when large enough roll towards the middle. There the liquid collects and coalesces to form a stream like film of water that is funneled to the tip and falls to the ground (Wigzell et al. 2016). There are difficulties in determining the transmission of intended target through food intake. Based on this, we used a nanomaterial-based delivery system to deliver dsRNA via body wall permeation in mites (Zheng et al. 2019).

**Fig. 2:**
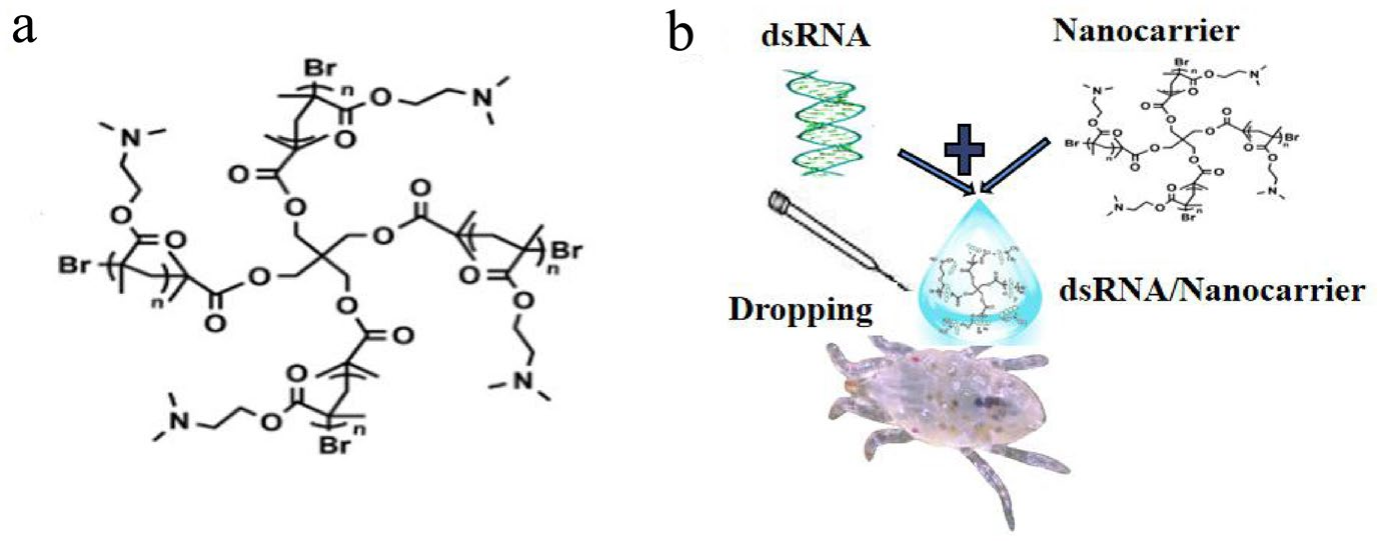
**a** Chemical structure of the nanocarrier SPc (from Li et al. 2019). **b** The dsRNA/nanocarrier transportation system

Gene vectors rationale is that polycations pack large gene coils into nanoparticles by charge neutralizing, which prevents gene degradation and facilitates its cellular uptake through endocytosis pathways (Ponnuswamy et al. 2017). The nanocarrier used is a star polycation (SPc) (Yan et al. 2019). Its star-shaped structure has a high density of functional groups and unique chemical properties of dendrimers (Fig. 2a), which are considered good for mass production of functional polymers. The chemical source of SPc, the monomer 2-N-(dimethyl aminoethyl) methacrylate (DMAEMA), is commercially available and an inexpensive chemical raw material (Li et al. 2019). In this study, for the first time we evaluate the gene-silencing effect and the lethal effect of dsRNA/nanocarrier on *S. nanjingensis*. Nanocarrier-based delivery systems not only have a simple operation procedure and high gene-silencing efficiency but also have low dsRNA consumption. This system was applied to *S. nanjingensis* for the first time, providing a new management method for mites. SPc is a kind of nanomaterial with high efficiency and low cost that can be applied in practical production (Yan et al. 2019).

## Materials and methods

### *Stigmaeopsis nanjingensis* rearing and experimental conditions

The original strain of *S. nanjingensis* was collected from the bamboo forest near the library of Guizhou University in Guiyang City, China, and kept in a climate control chamber, Institute of Entomology, Guizhou University. During the experiments, the bamboo leaves covered with wet cotton wool were placed in the culture dish for the feeding of *S. nanjingensis*. The culture conditions were 26 ± 0.5 °C, 70% RH, and L16:D8 photoperiod.

### Gene cloning and analysis of biological information

The fibroin gene was obtained by transcriptome sequencing of *S. nanjingensis*. Total RNA was extracted with MiniBEST Universal RNA Extraction Kit, following the manufacturer’s instructions (Takara Biomedical Technology, Beijing, China). First-strand cDNA was synthesized using StarScript II First-strand cDNA Synthesis Mix with gDNA Remover (Gene Star, Beijing, China), and RNA and cDNA concentrations were measured by a NanoDrop 2000c spectrophotometer. After quality checking, samples were stored at −80 and –20 °C for subsequent experiments.

All primers (Table S1) were designed by Primer v.5.0 software and synthesized by Sangon Biotech (Shanghai, China). The fibroin gene was amplified by using 2 × SanTaq PCR Master Mix (with Blue Dye) (Sangon Biotech, Shanghai, China). The PCR procedure was as follows: pre-denaturation at 94 °C for 4 min; 35 cycles of 94 °C for 30 s, 55 °C for 30 s and 72 °C for 1 min; and a final cycle of 72 °C for 10 min. The recovered purified product was ligated to the pGEM-T Easy vector (Promega, Beijing, China) and transformed into the *Escherichia coli* DH5α strain (Sangon Biotech). Positive colonies were selected and sent to Sangon Biotech for sequencing.

The cloned sequence was uploaded to the National Center for Biotechnology Information (NCBI) website (http://www.ncbi.nlm.nih.gov). Amino acid composition of the proteins was calculated using PredictProtein (https://predictprotein.org/). The transmembrane structure was calculated using the TMHMM Server (http://www.cbs.dtu.dk/services/TMHMM/). Hydrophobicity was calculated using ExPASy-ProtScale (https://web.expasy.org/protscale/). Signal peptide was calculated using SignalP v.3.0 (http://www.cbs.dtu.dk/services/SignalP/). Phosphorylation site prediction was carried out by using the NetPhos3.1 Server (http://www.cbs.dtu.dk/services/NetPhos/). Prediction of glycosylation sites was performed using the online software YinOYang v.1.2 (http://www.cbs.dtu.dk/services/Yin OYang/). The physicochemical properties were computed using the ExPASy-ProtParam tool (https://web.expasy.org/protparam/). Using the SOPMA tool, protein secondary structures were visualized (http://npsa-pbil.ibcp.fr/cgi-bin/npsa_automa t.pl?page = npsa_sopma.html).

### Relative expression of fibroin genes in different mite developmental stages

To investigate the expression profiles of fibroin, different developmental stages (egg, larva, nymph and adult) were collected with three biological replicates. In total 120 eggs, 100 larvae, 80 nymphs and 50 adults were collected for this experiment. The nine housekeeping genes (Actin, EF1A, RPL13, v-ATPase Tubulin, UBC, 28S rRNA, TBP, and 18S rRNA) were selected to design primers (Table S1) for screening the most stable housekeeping genes in different development stages, and their expression abilities in four developmental stages were evaluated by four algorithms (geNorm (Vandesompele et al. 2002), NormFinder (Andersen et al. 2004), BestKeeper (Pfaffl et al. 2004) and the ΔCt (Silver et al. 2006). All RNA was extracted by an RNA extraction kit (Takara Biomedical Technology) according to the manufacturer’s protocol. Integrity of RNA was determined by 1% agarose electrophoresis. The total RNA was reverse transcribed as described above.

The enzyme used in the qPCR was 2 × RealStar Green Fast Master Mix (Gene Star). The reaction conditions for the qPCR were as follows: 95 °C for 2 min, followed by 40 cycles of 95 °C for 15 s, 55 °C for 30 s, and 72 °C for 30 s. A melting curve was generated to ensure that no reaction produced nonspecific amplification. Relative standard curves for each step were generated from a fivefold cDNA dilution series. Using the 2^−∆∆Ct^ method, the gene expression levels were normalized to the abundance of the reference. Three technical repetitions were performed for each sample to determine the average value.

### Preparation of the dsRNA/nanocarrier formulation and the RNAi effect detection

Off target effects were detected using si-Fi tools (https://bio.tools/si-Fi). Synthesis of dsRNA was performed using the Transcript T7 High Yield Transcription Kit (Thermo Scientific, Shanghai, China). After synthesis, dsRNA was purified using the Gene JET RNA Purification Kit (Thermo Scientific). Nanomaterials and dsRNA were mixed gently at the recommended mass ratio of 1:1 (the final concentration of both nanomaterials and dsRNA was 500 ng/µl) to form a complex (Yan et al. 2019). The green fluorescent protein gene was used as a control (Thairu et al. 2017). Twenty female adult mites were placed onto a new leaf disc, and the dsFib/nanocarrier is dropped on the mite body wall by a microinjector (Narishige IM-31, Tokyo, Japan), at pressure 35 kPA. Amount of each injection was 20–50 nl (Fig. 2b). Treated leaf disc was placed in a climate chamber at 25 ± 1 °C with 70 ± 5% RH and L16:D8 photoperiod. As shown in Fig. 2b, female adult mites were treated with dsFib/nanocarrier, dsFib, dsGFP and nanocarrier, respectively. Each treatment consisted of 20 mites, three repetitions were used to determine the interference efficiency, and six repetitions were used to detect lethal effects. Time was recorded after the complete absorption of droplets, then three groups of droplets with the same volume were dropped on the blade, and their volatilization time was recorded (nine drops in each group).

After 24 h of treatment, RNA sample was extracted and reverse transcribed into cDNA. Gene interference efficiency was determined by quantitative PCR (qPCR) as described above. Morphological changes of silk were observed by electron microscopy.

### Statistical analysis

The 2^−∆∆Ct^ method was used for relative quantitation. The experimental data were analyzed by t-test in IBM-SPSS v.21.0 (IBM, Armonk, NY USA) to compare the difference between the treatment group and the control group. The expression pattern of the target gene was analyzed by the ANOVA in GraphPad Prism v.8.0 software. The software geNorm, NormFinder, BestKeeper and RefFinder were used to screen housekeeping genes. All column charts are made with GraphPad Prism v.8.0.

## Results

### Transcriptome sequencing

We separately completed the transcriptome sequencing of *S. nanjingensis*. In total, 6.41 Gb data were measured using the BGISEQ-500 platform (Fig. S1). 26,896 Unigenes were assembled and de-redundant. With BUSCO completeness scores (c) of 94% for *S. nanjingensis*, the Q20 base percentage exceeded 97.0%, the Q30 base percentage exceeded 92.4% (Table S2). Assembly of clean reads followed by clustering deredundant transcripts resulted in unigenes; 26,896 unigenes with an N50 of 2453 bp were generated (Fig. S2a). The transcriptome sequence data have been deposited in the Sequence Read Archive under BioProject ID PRJNA768242. UniGene functions were annotated to seven databases (Table 1).

**Table 1:** Statistical results of unigene annotation

Annotated database	No. unigenes	Percentage of all unigenes
NR	17,480	66.99
NT	6,728	25.01
Swiss-Prot	13,945	51.58
KEGG	14,765	54.90
KOG	13,542	50.35
Pfam	13,791	51.28
GO	11,309	42.05

*NR* non-redundant protein, *NT* nucleotide sequence database, *KEGG* Kyoto encyclopedia of genes and genomes, *KOG* euKaryotic orthologous groups, *Pfam* protein family, *GO* gene ontology

The highest two percentages of unigenes annotated were found in the NR and KEGG databases and were almost equivalent, with 67 and 55%, respectively. Moreover, the NT database had the lowest number of annotated unigenes with 6728 unigenes (25%). Based on the NR annotation information, a total of 17,480 unigenes have been annotated in the *S. nanjingensis* transcriptome, of which the most similar genes to *T. urticae* accounted for 81.8%, followed in order by *Limulus polyphemus* (0.91%), *Centruroides sculpturatus* (0.62%), *Dermatophagoides pteronyssinus* (0.55%), and *Tetranychus truncatus* (0.49%) (Fig. S2b).

Based on the GO database, functional classification statistics were performed on the transcriptome of *S. nanjingensis*, in which there were 7332 unigenes in the biological process, 9348 unigenes in the cellular component, 12,155 unigenes in total by molecular function (Fig. S2c). According to the annotation information of the KEGG database, a total of 14,765 unigenes were annotated onto six categories of metabolic pathways: cellular processes, environmental information processing, genetic information processing, human diseases, metabolism, and organismal systems (Fig. S2d).

### Cloning, characterization and expression patterns in different developmental stages of fibroin

**Fig. 3:**
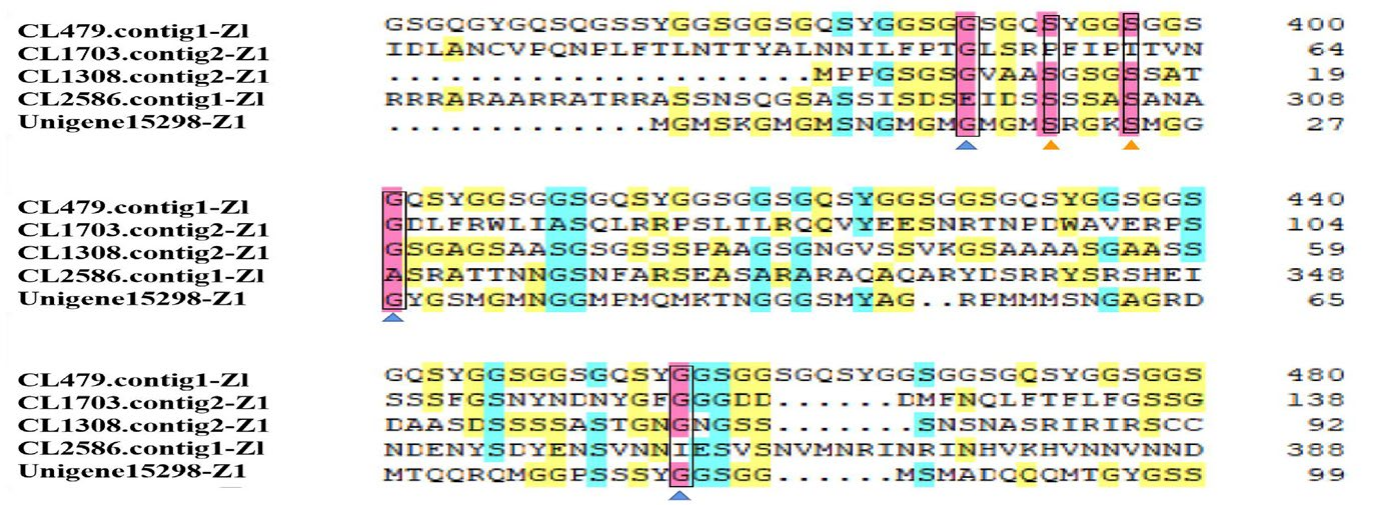
Alignment of candidate *Stigmaeopsis nanjingensis* fibroin. Amino acids with more than 75, 50 and 33% identity are in pink, green and yellow boxes, respectively. Blue and orange triangles indicate relatively conserved glycine and serine residues, respectively. (Color figure online)

In this study, 17 fibroins were identified in the transcriptome. Eight of the fibroins presented a complete ORF. Of these fibroins, the Unigene15321-Z1 and CL1353.contig8-Z1 have neither found the blast query nor structural features of fibroin. Both, CL1308.contig2-Z1 and CL2865.contig1-Zl found the blast query for the insect fibroin gene, the identity was between 67 and 74%. The CL1703.contig2-Z1 and Unigene15298_Z1 found the blast query for *T. urticae* fibroin gene, the identity was between 67 and 74%. The CL479.contig1-Zl has typical fibroin gene motif and a general motif of spider silk protein. The multiple alignment results are shown in Fig. 3. We found the composition similar to other fibroins, all with relatively conserved glycines, and comprising dipeptide repeating units composed of glycine-alanine or glycine-serine elements of low complexity. The ORF of target fibroin (CL479.contig1-Zl) was obtained from transcriptome data and verified by PCR. The sequence can be obtained in GenBank under accession number MZ436653. The complete ORF of the fibroin gene was 2493 bp long, encoding a total of 831 amino acids (Fig. S3), considered a heavy-chain component. The fibroin protein sequence contains 20 amino acids, among which glycine, serine and alanine account for > 50% of the total composition (Fig. S4a). The protein has no obvious transmembrane structure, so it was preliminarily identified as a non-transmembrane protein (Fig. S4b). Hydrophobic and hydrophilic residues are distributed throughout the polypeptide chain of fibroin, but the proportion of hydrophobic amino acids is lower than that of hydrophilic amino acids, so fibroin is considered a hydrophilic protein (Fig. S4c), and there are also signal peptide sites between amino acids 18 and 19 (Fig. S4d).

The secondary structure of fibroin consisted of an alternating pattern of random coils and α-helix and β-chain motifs (Fig. S4e), fitting the known fibroin secondary structure motifs. And the domain composition of *S. nanjingensis* fibroin protein has a typical fibroin motif. Two internal domains (domain 1: 330–485 and domain 2: 541–670), with distinct repetitive motifs (forming β-sheets), are separated by spacer domain (485–541). And there is a motif GGX (X: Ala, Ser), which is a general motif in spider silk protein (Table S3; Jelinski 1998). The unique toughness of these biopolymers is due to the secondary structure forming alternating crystalline (rigid) and amorphous (flexible) blocks (Sarkar et al. 2019). Fibroin has multiple phosphorylation and glycosylation sites. The calculated theoretical isoelectric point is 5.78, and the molecular weight is estimated to be 80.6 kDa.

**Fig. 4:**
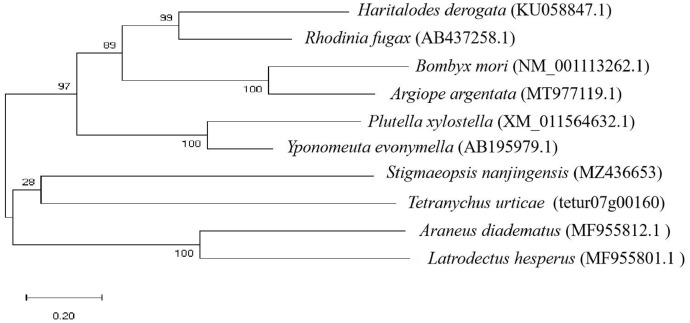
Evolutionary tree based on amino acid sequence of silk fibroin gene in arthropods. The phylogenetic tree was calculated using the Mega software with the maximum likelihood method

The sequence obtained in this study was compared with those of some silking arthropods recorded in the NCBI database, the results showed that the homology of fibroin gene in the arthropods was not high—even in the same family (Tetranychidae) it is not conservative (Fig. 4). By NCBI blast analysis, it was found that its sequence did not show strong homogeneity with the silk fibroin gene sequences of other arthropods. It is possible that the conservation of the silk protein gene in arthropods is not strong or that the fibroin of mites has a unique specificity.

**Fig. 5:**
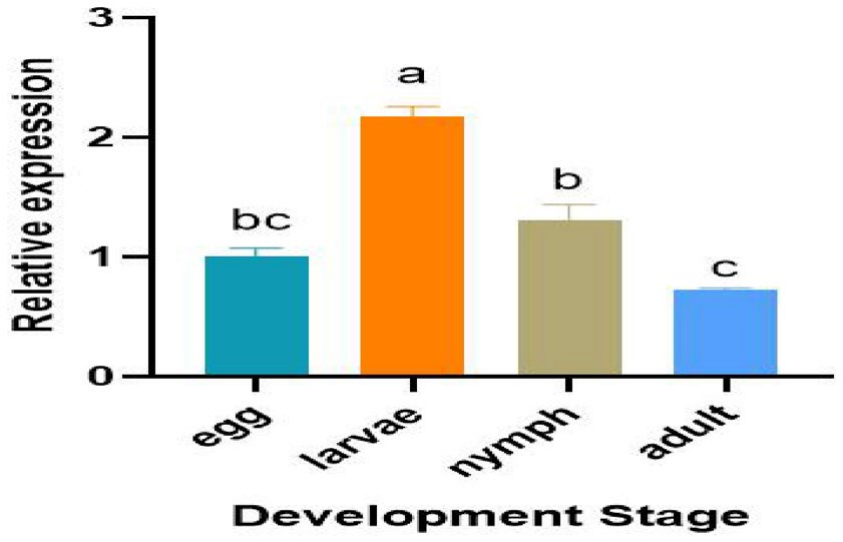
Mean (± SEM) relative expression of fibroin in four developmental stages of *Stigmaeopsis nanjingensis*. Means capped with different letters are significantly different (Tukey’s HSD test: P < 0.05)

Standard curves were generated for Fib and the nine housekeeping genes, using fivefold serial dilution of pooled cDNA. The correlation coefficient (R^2^) and PCR efficiency (E%) for each standard curve are shown in Table S4. R^2^ is 0.98–1.0, and efficiency of RT-qPCR is 90–110%. Using geNorm analysis, overall ranking of reference genes from most to least stable was: UBC > EF-1α > 18 s RNA > TBP > α-Tubulin > RPL13 > β-actin > 28 s RNA > v-ATPase (Fig. S5). The genetic stability ranking of BestKeeper analysis was significantly different from the other three methods. 18 s RNA, UBC, and EF-1α were all ranked in the top three in the stability analysis of ΔCt, geNorm, and NormFinder, whereas TBP was ranked first in the BestKeeper calculations. Comprehensive ranking calculated by RefFinder is: UBC > 18 s RNA > EF-1α > β-actin > 28 s RNA > Tubulin > RPL13 > v-ATPase > TBP (Table S5). It turned out that the most stable housekeeping gene in different developmental stages of *S. nanjingensis* was UBC. Therefore, UBC was selected as the housekeeping gene in this study. Relative expression of the fibroin gene was measured by qPCR after sampling each state of *S. nanjingensis* (Fig. 5). Results showed that expression level of fibroin varied greatly among mite stages. Expression in larvae was relatively high, followed by nymphs, and expression in eggs and adults was relatively low.

### Effects of dsFib/nanocarrier complexes on mite survival rate

**Fig. 6:**
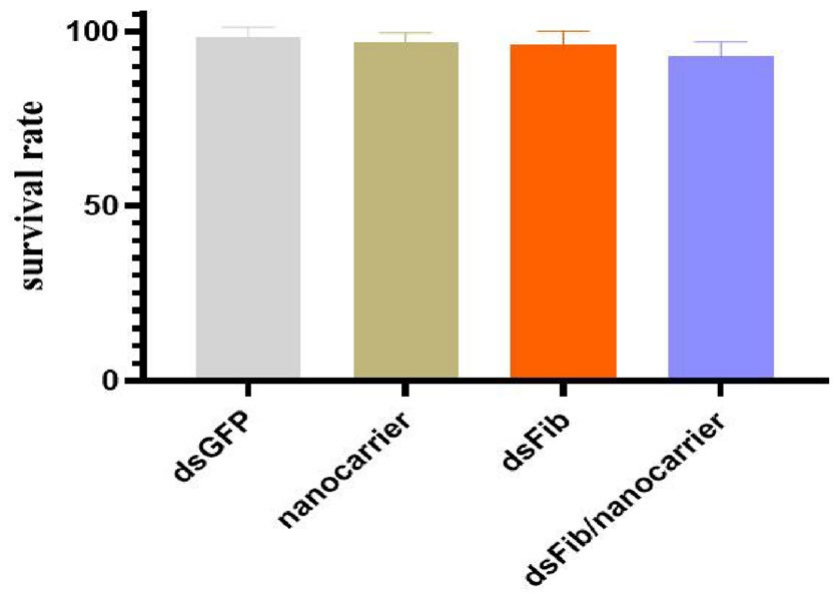
The effect of introducing nanocarrier on the survival rate of *Stigmaeopsis nanjingensis* can be neglected. Under the interference concentration of 500 ng/μl, the mean (± SEM) survival rates of *S. nanjingensis* treat with dsGFP, nanocarrier and dsFib/nanocarrier were > 92%. Means did not differ significantly (t-test: P > 0.05)

To verify the safety of nanocarriers for *S. nanjingensis*, we explored the lethal effects of nanocarriers and dsFib/nanocarrier composites on *S. nanjingensis*. Survival rate was 98.4% after 24 h of dsGFP treatment, 95.9% after nanocarrier treatment, 96% after dsFib treatment, and 92.8% after dsFib/nanocarrier complex treatment (Fig. 6). There was no significant difference among the three groups, which indicated that the nanocarrier was safe for *S. nanjingensis*.

### Detection of silence effect of dsFib/nanocarrier

**Fig. 7:**
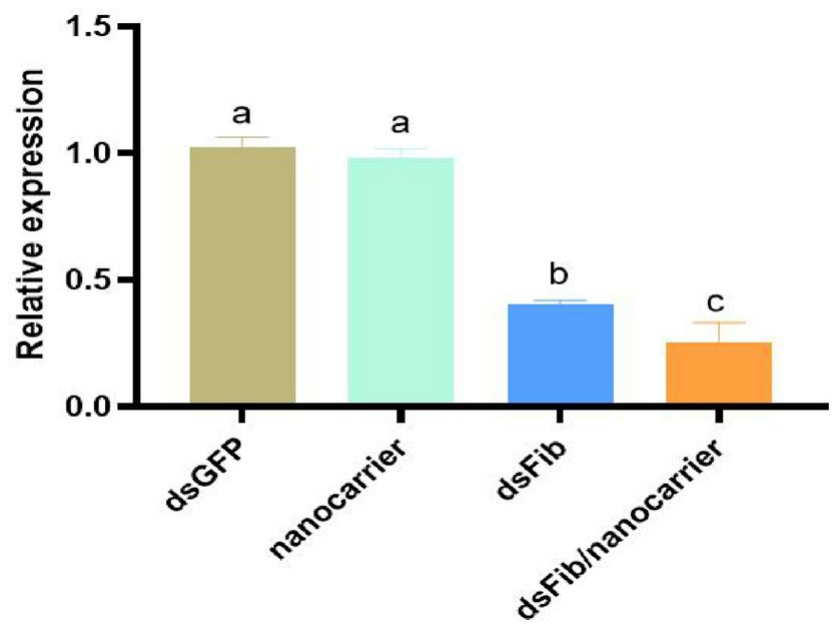
The mean (± SEM) expression of fibroin gene of the *Stigmaeopsis nanjingensis* is remarkably reduced after RNAi. Means capped with different letters are significantly different (t-tests for pairwise comparisons: P < 0.05)

The dsFib/nanocarrier droplets naturally adhered to the dorsa of *S. nanjingensis* and absorbed within 2 min, nanocarriers also absorbed in mites within 2 min, and average permeation time of dsGFP and dsFib is 2 min 14 s, whereas the average evaporation time of droplets with the same volume is 7 min 10 s. Our results indicate that all treatment groups penetrate into the body (Fig. S6). To determine the RNAi delivery system efficiency, fibroin gene levels were determined by RT-qPCR after 24 h of treatments. There was no significant difference between the nanocarrier and dsGFP control groups (Fig. 7). In dsFib/nanocarrier treatment, fibroin gene expression decreased by 75.4%, and interference efficiency of dsFib treatment group was 59.8%. synergized nanocarriers 15.6%. These results indicated that dsRNA and dsFib/nanocarrier complex effectively inhibit the expression of target genes, but interference efficiency of the dsFib/nanocarrier treatment group was significantly higher than that of the dsRNA treatment group. This shows that addition of nanocarriers improves the interference efficiency. The findings provide a new RNAi method for Tetranychidae and a pest control delivery system that could be applied in practical fields.

### Effect of gene interference on silk morphology of *Stigmaeopsis nanjingensis*

**Fig. 8:**
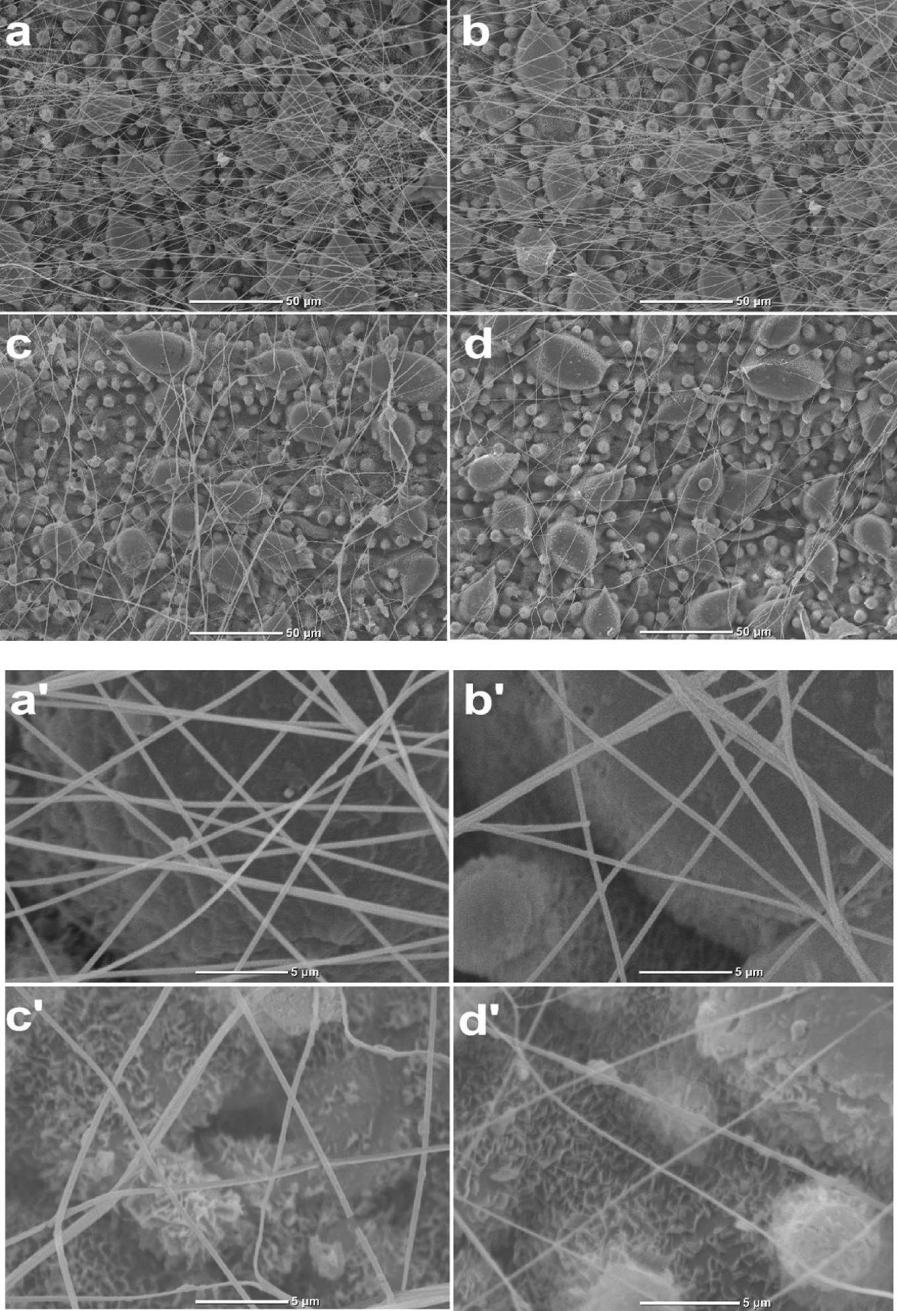
Actual electron micrographs of female adult silk of *Stigmaeopsis nanjingensis* after RNAi by body wall permeation based on nanocarriers. (**a**–**d, a'**–**d'**) Silk produced by female adult *S. nanjingensis* under the treatments of dsGFP, nanocarrier, dsFib and dsFib/nanocarrier groups, at (**a**–**d**) 500× and **(a'**–**d'**) 5000×

Comparison of scanning electron microscopy showed that morphological structure of the silk was not different between the two control treatments (Fig. 8). Silk in the dsGFP and nanocarrier treatment groups was clearly smoother and tighter than dsFib/nanocarrier treatment group, and net structure is neat and orderly. In dsFib and dsFib/nanocarrier treatment group, silk structure has uneven thickness, crimp and irregularity. The diameter of silk was generally smaller in the treated compared to the control group, and network structure was disorganized. The morphological structure of *S. nanjingensis* silk infiltrated with dsFib was damaged up to some extent after interference. This shows that fibroin may be related to the silk spinning behavior of *S. nanjingensis*.

## Discussion

Fibroin has traditionally been used to make textiles. Increasing research has shown that fibroin has important value in daily life and scientific research. Fibroin has good biocompatibility, material universality, mechanical resistance and controllable degradability; it can be used in artificial skin, blood vessels, bones materials as a suture in surgery and in biomedical materials (Barrett et al. 2013). At present, research on silk-producing arthropods is mainly focused on economic insects and spiders for the purpose of economic perspectives and national defense. However, many insects caused great loss to cash crops and ornamental plants by silk production. For example, *S. derogata* curle and eat cotton leaves, consequently the cotton plants cannot blossom and normally bear bolls, which seriously affects cotton production (Tang 2016). *Tetranychus urticae* has a strong reproductive capacity, and a large number of individuals spread by spinning silk and relying on wind, causing a sustained increase in the population and a wide range of hazards (Fleschner et al. 1956). *Stigmaeopsis nanjingensis* also has a strong silk spinning ability, which also increases the difficulty of biological and chemical control. Therefore, a new method with low environmental pollution risks has become an urgent requirement for the control of *S. nanjingensis.*

Most research on *S. nanjingensis* has focused on classification and biological habits, and less attention has been paid on molecular biology. Based on integral role play by silk in the life cycle of *S. nanjingensis*, we screened a fibroin gene through transcriptome information to explore the impact on silk production and functionality. In this study, we used silking performance to curb silking and reduce harm to target crops. The findings are expected to provide a new way to control silk-producing pests. Our results obtained from transcriptome data showed that fibroin genes are not conservative with each other in *S. nanjingensis*, that are similar to *T. urticae*. An evolutionary tree of other arthropods with fibroin genes, showed that different forms of fibroin lack homology (Numata 2020), even compared to *T. urticae*. However, it has characteristics that are similar to other arthropod fibroins, such as repeat motifs and amino acid composition. Secondary structure is a repeating pattern of alternating random coils and α-helix and β-chain motifs. In amino acid composition, serine, glycine and alanine account for the highest proportion (> 50%) of the amino acid composition, similar amino acid compositional bias is observable in silkworm fibroin. Subsequently, we explore its function through RNAi technology.

When using RNAi technology to control pests, effective introduction of dsRNA synthesized in vitro into insects is one of the strongest limiting factors. Different arthropods require appropriate dsRNA delivery systems to ensure efficient gene silencing and ease of manipulation (Zhang et al. 2013; Vogel et al. 2019). In spider mites, methods of dsRNA delivering include feeding method, soaking method and transgenic plants (Suzuki et al. 2017b). In this research, dsRNA was transmitted by the body wall permeation method using nanomaterials as the carrier. The nanocarrier used in this study is SPC, and its toxicity to *S. nanjingensis* is negligible. The SPc is a cationic dendrimer that condense random nucleic acids into complexes that are taken up through endocytosis (Win and Feng 2005). High-density amine functional groups at the periphery of nanocarrier enhance the affinity of complex for barrier components e.g., epidermis layer of insect body wall and cell membrane, body wall of Tetranychidae mainly composed of upper epidermis, outer epidermis, inner epidermis and dermatocyte layer (Hong et al. 1994), that are easily penetrated into the body compared to insects. In addition, SPc rapidly internalized into living cells with high efficacy of gene delivery and low cytotoxicity (Li et al. 2019). In our study, dsRNA/nanocarrier dose was placed on the dorsa of *S. nanjingensis* by a microinjector, the interference efficiency of dsFib/nanocarrier treatment was 75.4%, whereas dsFib treatment was 59.8%—addition of nanocarrier improved the interference efficiency (Fig. 6). As compared to common feeding methods, the amount of dsRNA required for a nanocarrier-based transdermal dsRNA delivery system is particularly low. In this study, 20–50 nl dsFib per *S. nanjingensis* and 2–4 uL dsRNA was sufficient for one treatment. During feeding method, a large amount of dsRNA was consumed without successful interference. Nanocarrier-based transdermal dsRNA delivery system reduces the consumption of dsRNA and saves the experimental cost. Electron microscopy results showed that silk in the control group was smooth and tight, with good fullness. However, after inhibition of the fibroin gene, the silk threads showed varying thickness, messy network structure, and the network gap became larger. Our results showed that fibroin gene played an important role in the silk formation process, and dsRNA targeting *S. nanjingensis* fibroin effectively affect the surface morphology of the net and flatness of the silk. How it affects the silk morphology remains to be further studied and analyzed. Moreover, web-spinning behavior is a complex trait controlled by multiple genes, in order to better explain the mechanism of silk spinning harm for *S. nanjingensis* and to curb the harm of silk spinning in application, there is still need much work to be carried out.

At present, pesticides are mainly used to control pests, but excessive use of chemical pesticides has caused various environmental problems, and long-term use has also made pests resistant (Gavrilescu et al. 2015). Nanocarriers carry dsRNA and pesticides, the delivery system reduces the use of pesticides and relieves the pressure of ecosystems on organisms (Xu et al. 2014a, b). However, RNAi is mainly used for the management of small arthropod pests, which have difficulties in determining the feed, are small in size, have weak body walls and are equipped with piercing-sucking mouthparts (Zheng 2018). The outermost wax layer on the body wall of arthropods is relatively thick—e.g., as in aphids—and it is necessary to add detergent to dsRNA/nanocarrier complexes to form dsRNA/nanocarrier/detergent complexes (Shen et al. 2014). The surfactant molecule in the detergent is amphiphilic, with hydrophilic groups forming a protective layer around the surface of the dsRNA/nanocarrier complex and lipophilic groups facing outward for subsequent adsorption to the insect body wall. The whole process only needs a simple operation by dropping, and high mortality and systematic errors caused by mechanical damage can be avoided, which provides an efficient and convenient operation scheme for scientific researchers and a new RNAi technology for mites. Thus, the nanocarrier-based transdermal dsRNA delivery system provides a new approach for the management of mite pests.

## Conclusion

This work explored a new control method from the perspective of biological silk production, mining fibroin protein genes of *S. nanjingensis* and using a body wall infiltration method based on nanomaterials for gene interference. Results showed that dsRNA/nanocarrier enters the mite body within 2 min after being dripped on the surface, and silencing efficiency is 75.4%. Interference efficiency is increased by 15.6% compared with direct dropping dsRNA. After interference, morphological structure of silk is obviously damaged. Our experimental results showed that interfering with the expression of the female adult fibroin protein gene by using nanomaterials may represent a new method for the prevention and control of *S. nanjingensis*. This study provides a research basis for elucidating the mechanisms of silk laying, cocoon formation and transfer damage in many agricultural and forest pests.

## Supplementary Information

Below is the link to the electronic supplementary material.

Supplementary file1 (PDF 150 KB)

Supplementary file2 (PDF 326 KB)

Supplementary file3 (PDF 89 KB)

Supplementary file4 (PDF 268 KB)

Supplementary file5 (PDF 100 KB)

Supplementary file6 (PDF 164 KB)

Supplementary file7 (PDF 108 KB)

Supplementary file8 (PDF 138 KB)

Supplementary file9 (PDF 90 KB)

Supplementary file10 (PDF 80 KB)

Supplementary file11 (PDF 81 KB)
