# Supplementary figures and images for: Identification of the fibroin of Stigmaeopsis nanjingensis by a nanocarrier-based transdermal dsRNA delivery system
Source: Exp Appl Acarol. 2022 May 11;87(1):31–47. doi: 10.1007/s10493-022-00718-7 (PMC9287230; doi:10.1007/s10493-022-00718-7)

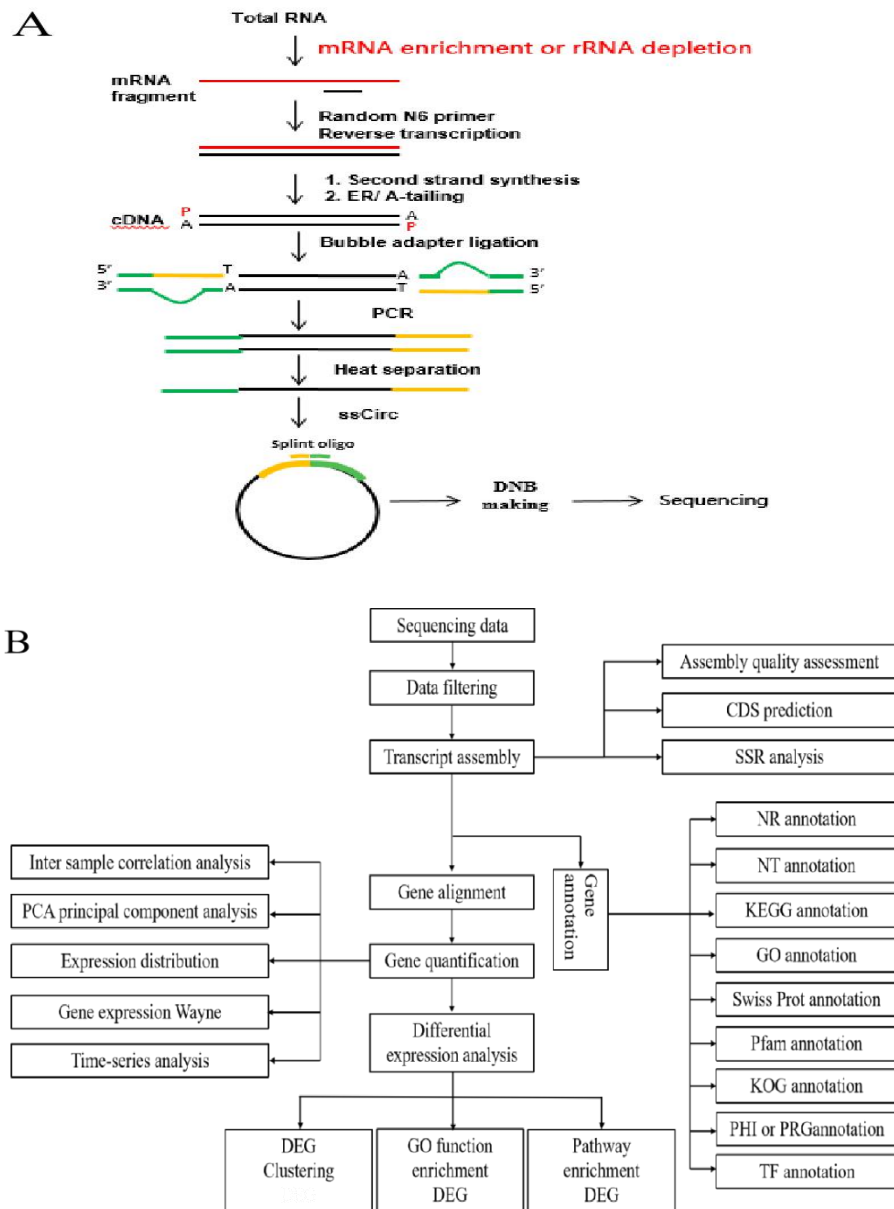

**Fig S1. (A) Construction of mRNA Library. (B) Flowchart of information analysis.**

Supplement: Supplementary file 1 — Supplementary file1 (PDF 150 KB) [file 10493_2022_718_MOESM1_ESM.pdf]
