# Supplementary material for: Identification of the fibroin of Stigmaeopsis nanjingensis by a nanocarrier-based transdermal dsRNA delivery system
Source: Exp Appl Acarol. 2022 May 11;87(1):31–47. doi: 10.1007/s10493-022-00718-7 (PMC9287230; doi:10.1007/s10493-022-00718-7)
Supplement: Supplementary file 2 — Supplementary file2 (PDF 326 KB) [file 10493_2022_718_MOESM2_ESM.pdf]

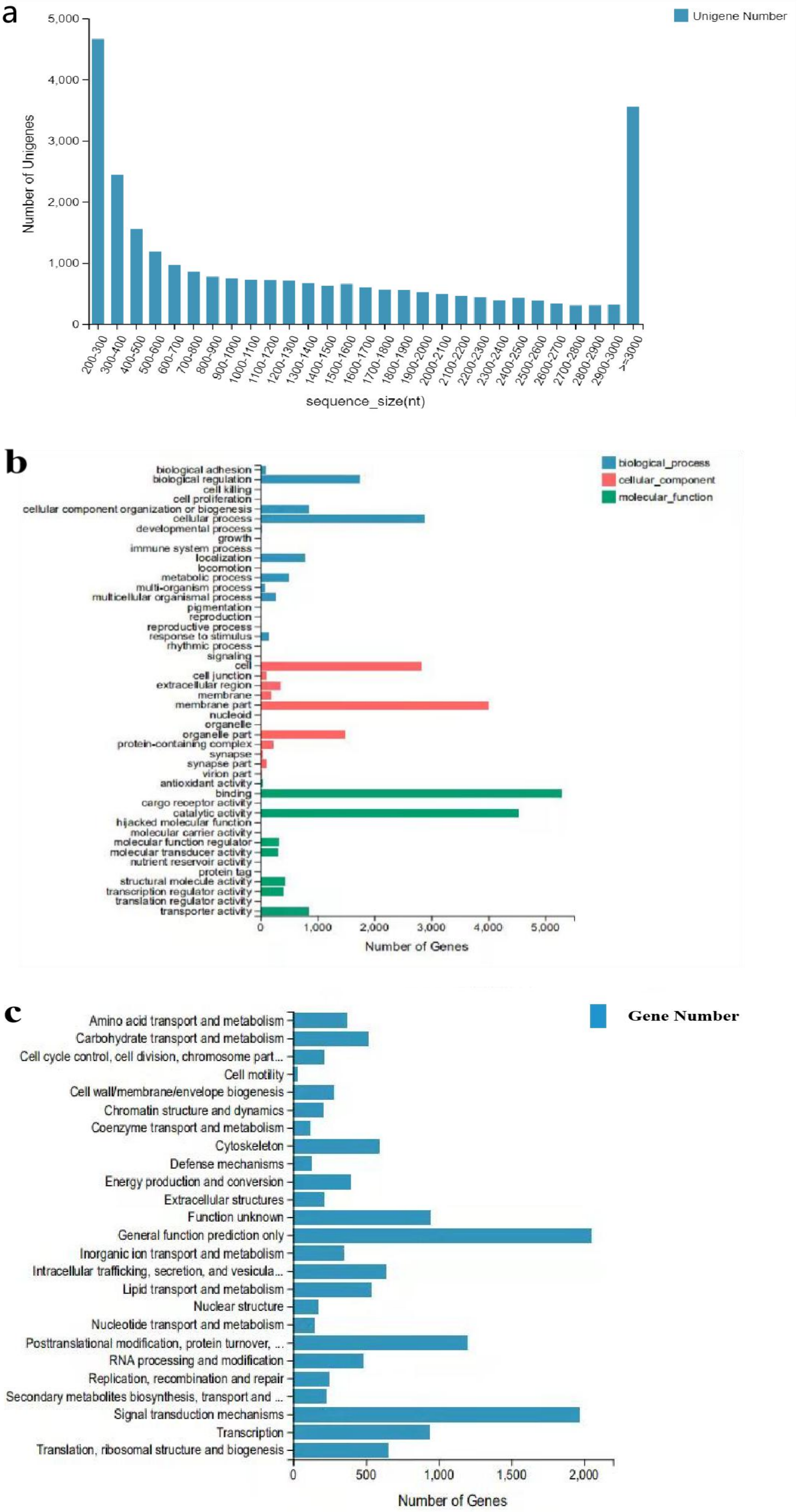
a
Unigene Number
Number of Unigenes
5,000
4,000
3,000
2,000
1,000
0
200-300
300-400
400-500
500-600
600-700
700-800
800-900
900-1000
1000-1100
1100-1200
1200-1300
1300-1400
1400-1500
1500-1600
1600-1700
1700-1800
1800-1900
1900-2000
2000-2100
2100-2200
2200-2300
2300-2400
2400-2500
2500-2600
2600-2700
2700-2800
2800-2900
2900-3000
>=3000
sequence_size(nt)
b
biological_process
cellular_component
molecular_function
biological adhesion
biological regulation
cell killing
cell proliferation
cellular component organization or biogenesis
cellular process
developmental process
growth
immune system process
localization
locomotion
metabolic process
multi-organism process
multicellular organismal process
pigmentation
reproduction
reproductive process
response to stimulus
rhythmic process
signaling
cell
cell junction
extracellular region
membrane
membrane part
nucleoid
organelle
organelle part
protein-containing complex
synapse
synapse part
virion part
antioxidant activity
binding
cargo receptor activity
catalytic activity
hijacked molecular function
molecular carrier activity
molecular function regulator
molecular transducer activity
nutrient reservoir activity
protein tag
structural molecule activity
transcription regulator activity
translation regulator activity
transporter activity
0
1,000
2,000
3,000
4,000
5,000
Number of Genes
c
Gene Number
Amino acid transport and metabolism
Carbohydrate transport and metabolism
Cell cycle control, cell division, chromosome part...
Cell motility
Cell wall/membrane/envelope biogenesis
Chromatin structure and dynamics
Coenzyme transport and metabolism
Cytoskeleton
Defense mechanisms
Energy production and conversion
Extracellular structures
Function unknown
General function prediction only
Inorganic ion transport and metabolism
Intracellular trafficking, secretion, and vesicula...
Lipid transport and metabolism
Nuclear structure
Nucleotide transport and metabolism
Posttranslational modification, protein turnover, ...
RNA processing and modification
Replication, recombination and repair
Secondary metabolites biosynthesis, transport and ...
Signal transduction mechanisms
Transcription
Translation, ribosomal structure and biogenesis
0
500
1,000
1,500
2,000
Number of Genes

d

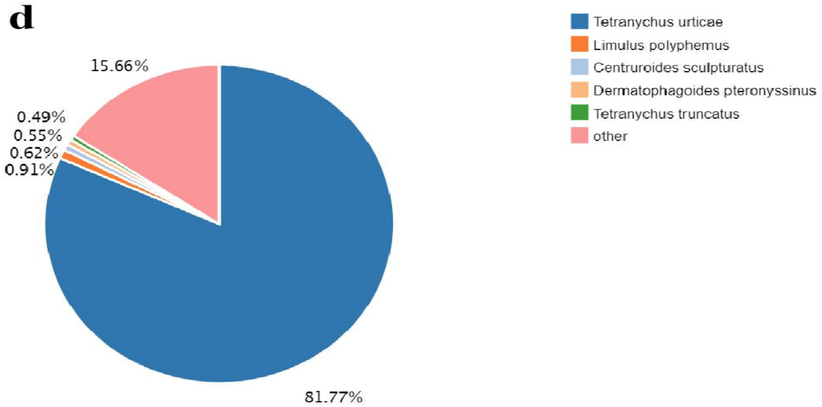

Fig S2. (a) Length distribution of all assembled unigenes in the antennal transcriptome of *S. nanjingensis*, (b)GO function distribution statistics, (c) KEGG function distribution statistics,(d) NR annotated species distribution.
