# Supplementary material for: Identification of the fibroin of Stigmaeopsis nanjingensis by a nanocarrier-based transdermal dsRNA delivery system
Source: Exp Appl Acarol. 2022 May 11;87(1):31–47. doi: 10.1007/s10493-022-00718-7 (PMC9287230; doi:10.1007/s10493-022-00718-7)
Supplement: Supplementary file 3 — Supplementary file3 (PDF 89 KB) [file 10493_2022_718_MOESM3_ESM.pdf]

1 atg tcc tta atc ggt ttg gtg ttt acc att tta caa atc agc cag gtt tgg tcg att ggt

1 M S L I G L V F T I L Q I S Q V W S I G

61 cct aag ttt aac tcc ttt aat caa caa ttt tcg gaa agt gat cta acg act tta cga gaa

21 P K F N S F N Q Q F S E S D L T T L R E

121 tta ttt gcc aaa gat aaa ctt ggt aac ggt tta aaa cga tcg gcg acc gtt aac caa gga

41 L F A K D K L G N G L K R S A T V N Q G

181 ggt ttt gga ggg ccc att gca gct tcg tca aac aat caa gcc tcc att gtt gtc ctt ccg

61 G F G G P I A A S S N N Q A S I V V L P

241 ttg tac ctt cgt aac aaa aat cag gct aaa acg cca atc ttt tac gat gct att caa gg

81 L Y L R N K N Q A K T P I F Y D A I Q G

301 gct gaa agt tca ggt aaa gtt gcc aga gca cct ctc gta gct gcc gct tca cca gtc tca

101 A E S S G K V A R A P L V A A A S P V S

361 cat tac gga tct tca cca tcc cta aca tct tca tcg cca gct gtt tca tct tca tca ccg

121 H Y G S S P S L T S S S P A V S S S S P

421 tca gcc tca tct tca gta gct cca tca gct gga aga aaa agt gag tta act cga tcg gag

141 S A S S S V A P S A G R K S E L T R S E

481 gtg aat cgt tat tta gct gct cca tcc aga agt aat gtt gcc gtt tat ggt ggc tca tct

161 V N R Y L A A P S R S N V A V Y G G S S

541 aaa cca acc tca att caa cct gta tca cct tcg tct tcc gtt agt tcc tct ggt act tct

181 K P T S I Q P V S P S S S V S S S G T S

601 tat gga aga ccc gct gaa att tat cct gcc gag tct acg tca aat gga gcc tcc tct gtt

201 Y G R P A E I Y P A E S T S N G A S S V

661 ctt gcc caa gct ggt tac gct gct gct agt ccc aat tct cat ctt gct gtg gcc gct gct

221 L A Q A G Y A A A S P N S H L A V A A A

721 gct ggt tca ggt agc tct ggt tat ggc tca tct ggt ggc aaa cct acc tca tct ggt caa

241 A G S G S S G Y G S S G G K P T S S G Q

781 ggt gca tcg aca gtc cgt gga tac ccc gca ttg aag ccg tta cac gtt gaa gaa cac caa

261 G A S T V R G Y P A L K P L H V E E H Q

841 aag gaa gct ttt caa gat ttt acc gga gct ttg gag aat cat gaa ctt aaa cct ttg acc

281 K E A F Q D F T G A L E N H E L K P L T

901 aac caa gat gtt tac gat tta cca gcg ata cgt ccc ggt gat ttg act ggt gat tct gtt

301 N Q D V Y D L P A I R P G D L T G D S V

961 tca act aaa aaa caa gaa tct cga ggt tca caa aat caa gga ggt tac ggt gat gga gtt

321 S T K K Q E S R G S Q N Q G G Y G D G V

1021 tca tct caa gga aat gct tac gga tca aac tct aat caa ggt tac tca gga cgt caa gaa

341 S S Q G N A Y G S N S N Q G Y S G R Q E

1081 gga tca ggt caa gga tac ggt caa agc caa ggc tca tct tat ggt gga tca ggg gga tca

361 G S G Q G Y G Q S Q G S S Y G G S G G S

1141 gga caa tca tat ggt ggt tct gga ggc tca gga caa tca tac ggt gga tca ggc gga tca

381 G Q S Y G G S G G S G Q S Y G G S G G S

1201 gga caa tca tat ggt ggt tct gga ggc tca gga caa tca tac ggt gga tca ggt gga tca

401 G Q S Y G G S G G S G Q S Y G G S G G S

1261 gga caa tca tat ggt ggt tct gga ggc tca gga caa tca tat ggt gga tca ggt gga tca

421 G Q S Y G G S G G S G Q S Y G G S G G S

1321 gga caa tca tat ggt ggt tct ggt gga tca gga caa tca tac ggt gga tca ggc gga tca

441 G Q S Y G G S G G S G Q S Y G G S G G S

1381 gga caa tca tat ggt ggt tct gga ggc tca gga caa tca tac ggt gga tca ggt gga tca

461 G Q S Y G G S G G S G Q S Y G G S G G S

1441 gga caa tca tat ggt gct tct aat gac ggt caa tca tat ggt tca tcc aat gga cca agt

481 G Q S Y G A S N D G Q S Y G S S N G P S

1501 caa tct tac agt tca tca tct gga ggt tac gaa tca agc tca aat caa gga tac agc ggt

501 Q S Y S S S S G G Y E S S S N Q G Y S G

1561 gga tca gcg cct gcc agt gga tca tat gga caa ggt tct tct gcc cca aga caa aat agc

521 G S A P A S G S Y G Q G S S A P R Q N S

1621 tat ggc gga caa agt gcc act caa ggt tcc tat gga ggt caa tca ggt tcg tct gga ggt

541 Y G G Q S A T Q G S Y G G Q S G S S G G

1681 tat ggt gga tcg tct ggc tac ggt aac agc gga aac tct ggt tac gga gct tct aat tct

561 Y G G S S G Y G N S G N S G Y G A S N S

1741 caa tct tat ggt ggc caa tca ggg tct gct ggt ggt caa tca ggt tca act gga ggc tat

581 Q S Y G G Q S G S A G G Q S G S T G G Y

1801 ggt gga caa tcc gga ggt cat ggt gct tca tct ggc tat ggt aat agt aat tct ggt tac

601 G G Q S G G H G A S S G Y G N S N S G Y

1861 ggt gct tca gcc gct gct ggg gga tca ggt tat tct agt caa ggc tac ggt gct gca gcc

621 G A S A A A G G S G Y S S Q G Y G A A A

1921 gcc gct gcc gat aac agt gga gct tat acc gct agc gct gga gat tat gct act gca gca

641 A A A D N S G A Y T A S A G D Y A T A A

1981 gca gcc gca gca gct gta gac gct acc aca tac gga atc gga tcc acc ggg ccc att aac

661 A A A A A V D A T T Y G I G S T G P I N

2041 ccg tca tac ttg tcc tcc ttc cct att gaa acc acc tat ggt tca gca gca tct tca cca

681 P S Y L S S F P I E T T Y G S A A S S P

2101 atg ggc ttc cac caa tct atc ggc tca tca tct cct tat gcc tca gct gtt cct act tac

701 M G F H Q S I G S S S P Y A S A V P T Y

2161 gat gaa tct cct caa tca agc gcc atc ggt gac aca gaa tat ggc tca ggc tca gct cct

721 D E S P Q S S A I G D T E Y G S G S A P

2221 gtc tcc tca tac gaa ccc caa gct tca tca tcc tat gga tca tca tct ctc cca tct tca

741 V S S Y E P Q A S S S Y G S S S L P S S

2281 ttt tac tca aat gtt act cca tac agt caa tct gtt ggt caa gcc tgt gtg act ccc caa

761 F Y S N V T P Y S Q S V G Q A C V T P Q

2341 aat aac gct ggt tat ggt aca aat cca atc cat tca tcg acc tat aat caa aat gga tac

781 N N A G Y G T N P I H S S T Y N Q N G Y

2401 gaa aat aca tac gcc gct gcc tca tca cca caa tct gtt tcc tct cag gca cca gcc tca

801 E N T Y A A A S S P Q S V S S Q A P A S

2461 gct tct gtc tca gca acc gtc gat cag cgt tac tag

821 A S V S A T V D Q R Y *

Figure. S3 The ORF of Fibroin of *Stigmaeopsis nanjingensis*
