# Supplementary material for: Identification of the fibroin of Stigmaeopsis nanjingensis by a nanocarrier-based transdermal dsRNA delivery system
Source: Exp Appl Acarol. 2022 May 11;87(1):31–47. doi: 10.1007/s10493-022-00718-7 (PMC9287230; doi:10.1007/s10493-022-00718-7)
Supplement: Supplementary file 4 — Supplementary file4 (PDF 268 KB) [file 10493_2022_718_MOESM4_ESM.pdf]

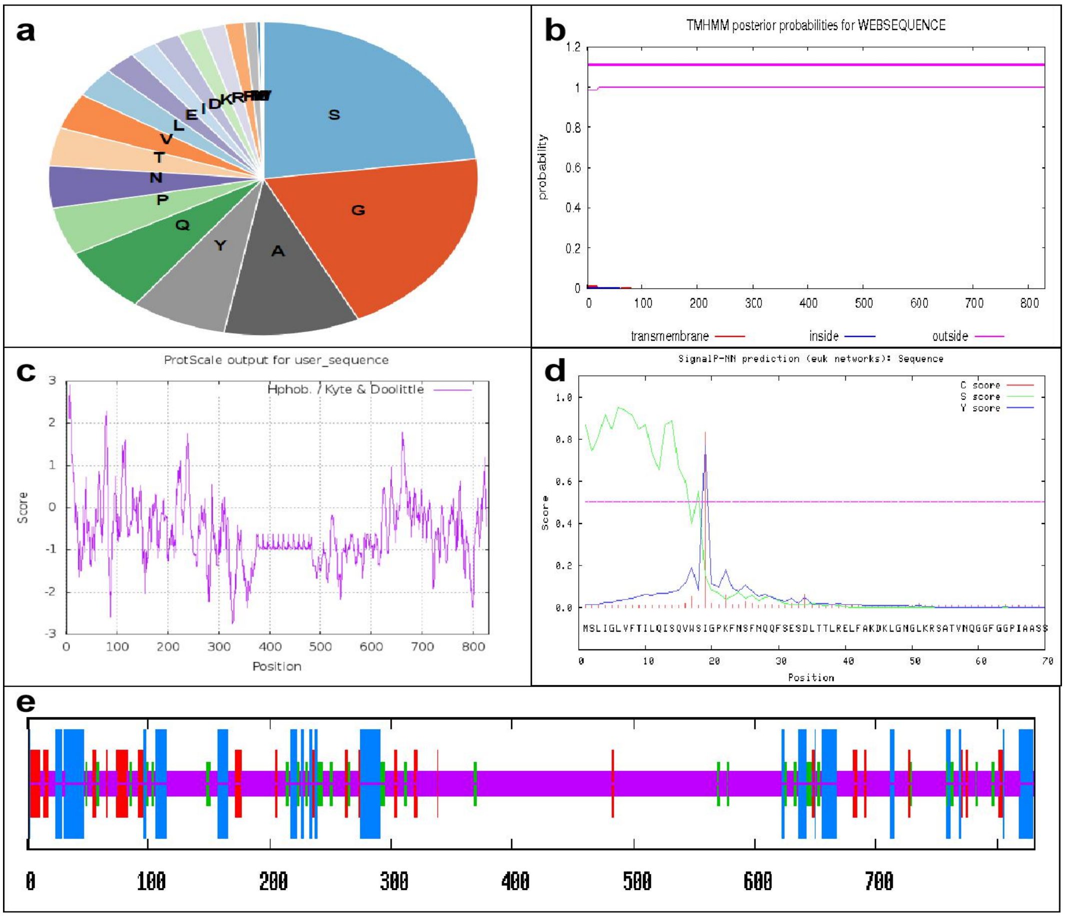

Fig. S4 Bioinformatics analysis of fibroin **(a)** Amino acid composition of fibroin. **(b)** Transmembrane structure analysis. **(c)** Hydrophobicity analysis. **(d)** Signal peptide analysis. **(e)** Secondary structure of the fibroin, bule: α-helix, red: β-strand, gree: β-turn, purple: random coil
