# Supplementary material for: Identification of the fibroin of Stigmaeopsis nanjingensis by a nanocarrier-based transdermal dsRNA delivery system
Source: Exp Appl Acarol. 2022 May 11;87(1):31–47. doi: 10.1007/s10493-022-00718-7 (PMC9287230; doi:10.1007/s10493-022-00718-7)
Supplement: Supplementary file 5 — Supplementary file5 (PDF 100 KB) [file 10493_2022_718_MOESM5_ESM.pdf]

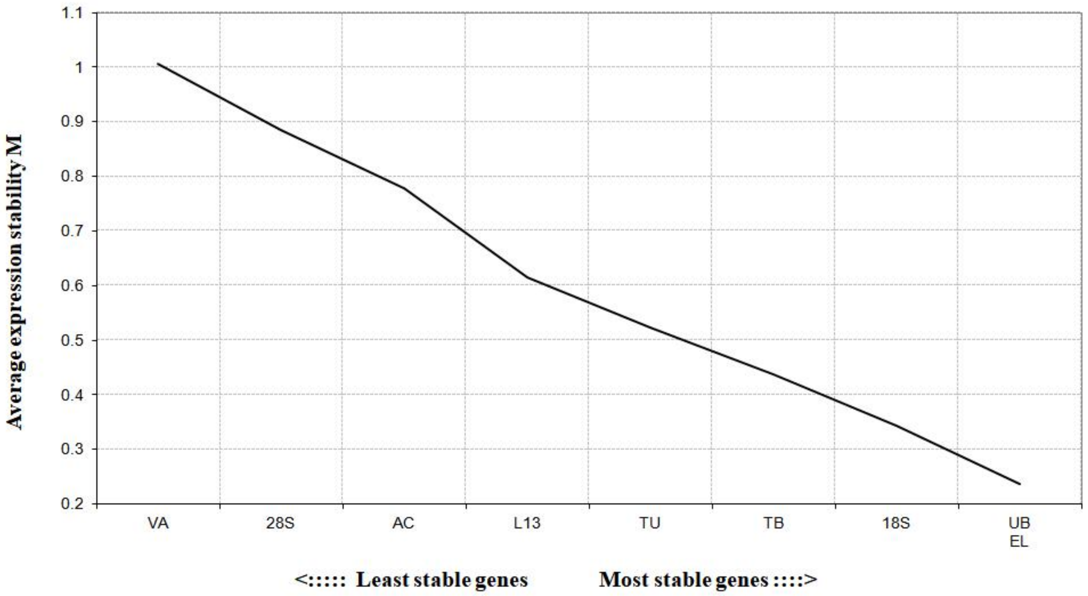

Fig. S5 Ranking of the 9 housekeeping genes based on the stability value (M).Using geNorm analysis, the smaller the M value, the more stable it is. UBC and EF-1α were listed as the most stable reference genes.
