# Supplementary material for: Identification of the fibroin of Stigmaeopsis nanjingensis by a nanocarrier-based transdermal dsRNA delivery system
Source: Exp Appl Acarol. 2022 May 11;87(1):31–47. doi: 10.1007/s10493-022-00718-7 (PMC9287230; doi:10.1007/s10493-022-00718-7)
Supplement: Supplementary file 6 — Supplementary file6 (PDF 164 KB) [file 10493_2022_718_MOESM6_ESM.pdf]

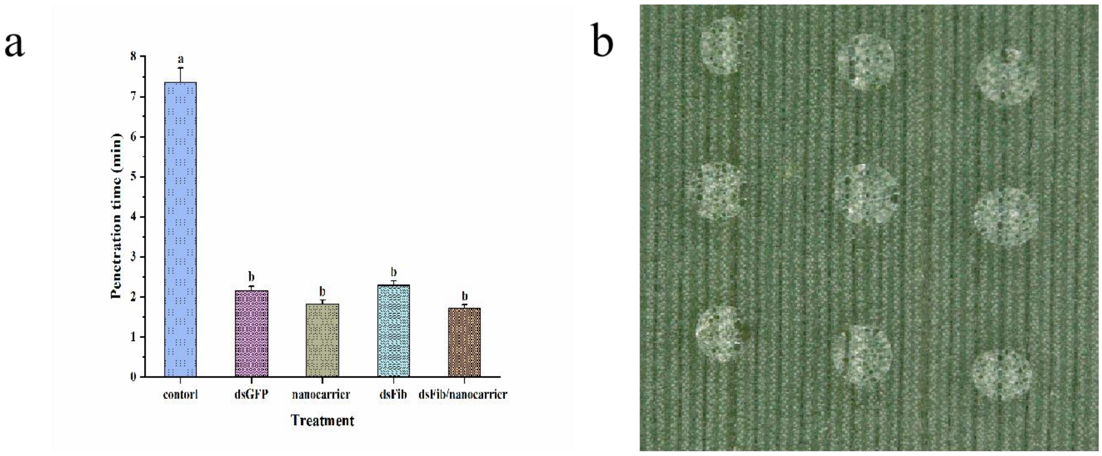

Fig. S6 (a) Time for different droplets to penetrate into the body wall, the control group is that natural evaporation time of the liquid drop (Tukey's HSD test, P < 0.05). (**b)** The liquid drop naturally evaporate on that bamboo leaves.
