# Supplementary material for: Identification of the fibroin of Stigmaeopsis nanjingensis by a nanocarrier-based transdermal dsRNA delivery system
Source: Exp Appl Acarol. 2022 May 11;87(1):31–47. doi: 10.1007/s10493-022-00718-7 (PMC9287230; doi:10.1007/s10493-022-00718-7)
Supplement: Supplementary file 7 — Supplementary file7 (PDF 108 KB) [file 10493_2022_718_MOESM7_ESM.pdf]

Table. S1 Primers used in this experiment

Primer name	sequence(5′–3′)
**Gene clone**	
Fib-H	1F: ATCTCCGTTATGTCTCCTCC
	1R: CTCCATCACCGTAACCTCCT
	2F: TCATCTCCGTTATGTCTCCTC
	2R: CGTATCGCTGGTAAATCGTA
	3F: ACCCGTCATACTTGTCCTCC
	3R: TTTTACCCATTGCTTCTTTG
**Quantitative real-time PCR**	
qFib-H	F: GCTGGAGATTATGCTACTGC
	R: AGCCGATAGATTGGTGGAA
qUBC	F: TCCTTCATAAAGAGTCCCAGA
	R: TCAATCCAGACTGAACCACC
**dsRNA synthesis**	
dSFib	F: TAATACGACTCACTATAGGGGCCTCCTCTGTTCTTGCC
	R: TAATACGACTCACTATAGGGCTCCATCACCGTAACCTCCT
dSGFP	F: TAATACGACTCACTATAGGGGCCAACACTTGTCACTACTT
	R: TAATACGACTCACTATAGGGGGAGTATTTTGTTGATAATGGTCG
**RT-qPCR primer of housekeeping gene.**	
*β-a*ctin	F:AGGTATCGCTGACAGAATGC
	R:TGGTCCAGATTCATCGTATTC
EF-1α	F: CCTGAGGAGTCCAACGAAC
	R: CAACAACTGGCAAAGCGTA
RPL13	F:TGTTGGTATGTCTGTTGGCTAT
	R:ACCCGTCTCGTGATTGTTAT
α-Tubulin	F:GGTGATGTAGTTCCCAAAGA
	R:AACGGTCGGTGGTTGATAG

v-ATPase	F:GCAGTGACTCGGGACTACAT<br>R:TCTCACAATCTCGGCATACA
28S rRNA	F:TCTTGGTTATTACTTCGGTGG<br>R:GAGAGACACTATCCCAACTTCC
TBP	F: TGGGTCTCAGCAGCAATC<br>R：TAATGACCGCAGCAAACC
18S rRNA	F:GCTAGTAGCCTGTTGGATGG<br>R:CATTCGCGGTTTCACTTT
UBC	F:TCAATCCAGACTGAACCACC<br>R:TCCTTCATAAAGAGTCCCAGA
