# Supplementary material for: Identification of the fibroin of Stigmaeopsis nanjingensis by a nanocarrier-based transdermal dsRNA delivery system
Source: Exp Appl Acarol. 2022 May 11;87(1):31–47. doi: 10.1007/s10493-022-00718-7 (PMC9287230; doi:10.1007/s10493-022-00718-7)

Table. S2 (A) Statistics of transcriptome sequencing results of *S.nanjingensis*. (B) Base quality distribution of clean reads.

(A)

SEQUENCING RESULTS	QUANTITY OR FREQUENCY
TOTAL RAW READS（M）	43.82
TOTAL CLEAN READS （M）	42.76
Total Clean Bases(Gb)	6.41
N PERCENTAGE (%)	0.45
Clean Reads Q20(%)	97.02
Clean Reads Q30(%)	92.39
Clean Reads Ratio(%)	97.57

(B)

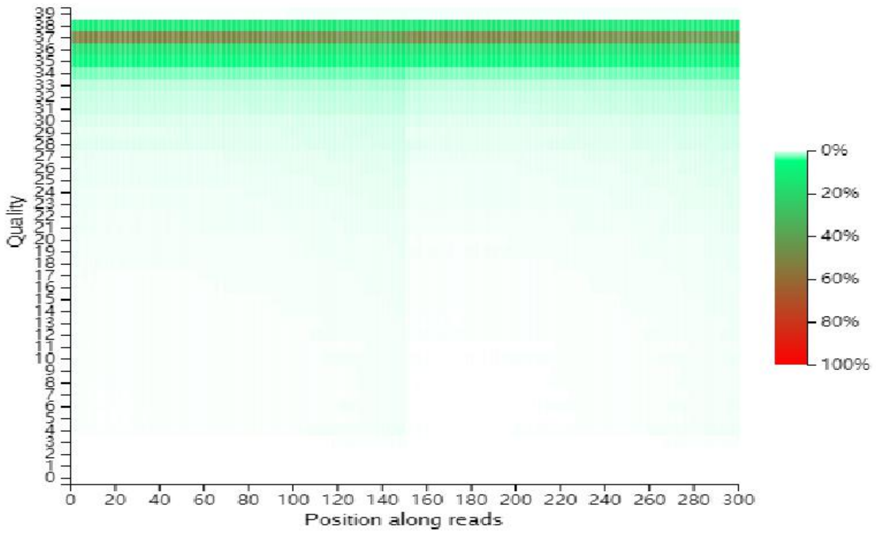

Supplement: Supplementary file 8 — Supplementary file8 (PDF 138 KB) [file 10493_2022_718_MOESM8_ESM.pdf]
