# Supplementary material for: Identification of the fibroin of Stigmaeopsis nanjingensis by a nanocarrier-based transdermal dsRNA delivery system
Source: Exp Appl Acarol. 2022 May 11;87(1):31–47. doi: 10.1007/s10493-022-00718-7 (PMC9287230; doi:10.1007/s10493-022-00718-7)
Supplement: Supplementary file 9 — Supplementary file9 (PDF 90 KB) [file 10493_2022_718_MOESM9_ESM.pdf]

Table. S3 Domain composition of *S. nanjingensis* fibroin protein.

Sequence of fibroin	Domain
MSLIGLVFTILQISQVWSIGPKFNSFNQQFSESDLTTLRELFAKDKLGNGLKRSATVNQGGFG<br>GPIAASSNNQASIVVLPLYLRNKNQAKTPIFYDAIQGAESSGKVARAPLVAAASPVSHYGSSPS<br>LTSSSPAVSSSSPSASSSVAPSAGRKSELTRSEVNRYLAAPSRSNVAVYGGSSKPTSIQPVSPSSS<br>VSSSGTSYGRPAEIYPAESTSNGASSVLAQAGYAAASPNSHLAVAAAAGSGSSGYGSSGGKPT<br>SSGQGASTVRGYPALKPLHVEEHQKEAFQDFTGALENHELKPLTNQDVYDLPAIRPGDLTGD<br>SVSTKKQESRGSQNQGGYGDGVSSQGNAYGSNSNQGYSGRQE	N-terminal domain
GSGQGYGQSQGSSYGGSGGSGQSYGGSGGSGQSYGGSGGSGQSYGGSGGSGQSYGGSG<br>GSGQSYGGSGGSGQSYGGSGGSGQSYGGSGGSGQSYGGSGGSGQSYGGSGGSGQSYG<br>GSGGSGQSYGASNDGQSYG	Internal domain 1
SSNGPSQSYSSSSGGYESSSNQGYSGGSAPASGSYGQGSSAPRQN	Spacer domain
SYGGQSATQGSYGGQSGSSGGYGGSSGYGNSGNSGYGASNSQSYGGQSGSAGGQSGSTG<br>GYGGQSGGHGASSGYGNSNSGYGASAAAGGSGYSSQGYGAAAAAADNSGAYTASAGDYA<br>TAAAAAAAVDATT	Internal domain 2
YGIGSTGPINPSYLSSFPIETTYGSAASSPMGFHQSIGSSSPYASAVPTYDESPQSSAIGDTEYG<br>SGSAPVSSYEPQASSSYGSSSLPSSFYSNVTPYSQSVGQACVTPQNNAGYGTNPIHSSTYNQ<br>NGYENTYAAASSPQSVSSQAPASASVSATVDQRY	N-terminal domain
