# Supplementary material for: Identification of the fibroin of Stigmaeopsis nanjingensis by a nanocarrier-based transdermal dsRNA delivery system
Source: Exp Appl Acarol. 2022 May 11;87(1):31–47. doi: 10.1007/s10493-022-00718-7 (PMC9287230; doi:10.1007/s10493-022-00718-7)
Supplement: Supplementary file 10 — Supplementary file10 (PDF 80 KB) [file 10493_2022_718_MOESM10_ESM.pdf]

Table. S4 Primer amplification efficiency.

Gene symbol	Gene name	R^2	E(%)*
*β-a*ctin	Beta actin	0.99	104%
EF-1α	Elongation factor 1 alpha	0.99	92%
RPL13	ribosomal protein L13	0.99	92%
α-Tubulin	Alpha tublin	0.98	105%
v-ATPase	vacuolar-type H+-ATPase	0.98	92%
28S rRNA	28S ribosomal RNA	0.99	96%
TBP	TATA-box-binding protein	0.98	105%
18S rRNA	18S ribosomal RNA	0.99	95%
UBC	Ubiquitin conjugating enzyme	0.99	100%
Fib	Fibroin	0.98	95.6%
