# Supplementary material for: Identification of the fibroin of Stigmaeopsis nanjingensis by a nanocarrier-based transdermal dsRNA delivery system
Source: Exp Appl Acarol. 2022 May 11;87(1):31–47. doi: 10.1007/s10493-022-00718-7 (PMC9287230; doi:10.1007/s10493-022-00718-7)
Supplement: Supplementary file 11 — Supplementary file11 (PDF 81 KB) [file 10493_2022_718_MOESM11_ESM.pdf]

Table. S5 Stability of reference gene expression at different developmental stages of *S. Nanjingensis.*

Reference gene	geNorm		NormFinder		BestKeeper		ΔCt	
	Stability	Rank	Stability	Rank	Stability	Rank	Stability	Rank
β-actin	0.73	5	0.665	4	1.333	8	1.095	4
EF1A	0.334	3	0.572	3	0.914	4	1.01	3
RPL13	1.095	8	1.172	7	0.991	5	1.424	7
α-Tubulin	0.612	4	1.059	6	1.427	9	1.281	6
v-ATPase	0.967	7	1.192	8	1.269	7	1.446	8
28S rRNA	0.802	6	0.721	5	1.093	6	1.123	5
TBP	1.195	9	1.339	9	0.78	1	1.545	9
18S rRNA	0.285	1	0.33	1	0.908	3	0.908	1
UBC	0.285	1	0.354	2	0.865	2	0.921	2
